# Exploring mechanisms of differential vulnerability in Early‐Onset vs. Late‐Onset sporadic Frontotemporal Dementia: a single‐center longitudinal study

**DOI:** 10.1002/alz70856_101977

**Published:** 2025-12-25

**Authors:** Chiara Giuseppina Bonomi, Emanuele Ginevra, Caterina Motta, Alessandro Martorana

**Affiliations:** ^1^ University of Rome Tor Vergata, Rome, Rome, Italy

## Abstract

**Background:**

Sporadic frontotemporal dementia (FTD) can affect both younger (<65 years old, Early‐Onset, eoFTD) and older individuals (≥65 years old, Late‐Onset, loFTD). The mechanisms behind varying onset ages are intriguing yet underexplored. This study aimed to investigate whether differences in neurodegeneration intensity, microglial activation, and vascular comorbidities distinguish eoFTD from loFTD or differently impact longitudinal cognitive decline, potentially elucidating mechanisms of different vulnerability in these subgroups.

**Method:**

52 consecutive patients with sporadic FTD, stratified according to age at onset (eoFTD, *n* = 28; loFTD, *n* = 24), underwent lumbar puncture with CSF dosage of amyloid‐β42, phosphorylated‐tau (*p*‐tau), total‐tau (t‐tau), albumin quotient (Qalb), neurofilament light chains (NfL), and soluble TREM‐2 (sTREM‐2). Eight vascular risk factors were computed in a cumulative vascular score (VS). First, we compared CSF levels of each biomarker and VS between eoFTD and loFTD. Then, we used multivariate regression analyses to verify the association of CSF NfL, sTREM‐2, VS and Qalb, with cognitive decline at 18±3 months follow‐up (ΔMMSE) in each group.

**Result:**

The groups were comparable in age, sex and in the prevalence of clinical FTD phenotypes (eoFTD: 21 bvFTD/8 PPA; loFTD: 18 bvFTD/6 PPA). We found no differences in CSF biomarkers and sTREM‐2, and a trend toward higher CSF NfL levels in loFTD compared to eoFTD (*p* = 0.061). Qalb and VS values were similar. Longitudinal evaluation further indicated that ΔMMSE were comparable between eoFTD and loFTD (*p* = 0.853). Multivariate regressions showed a negative association between NfL and ΔMMSE in the whole cohort (β=‐0.528, *p* = 0.005), with higher effect size in eoFTD (β=‐0.809, *p* = 0.011) but no significant association in loFTD. Age, Qalb values, sTREM‐2 levels and VS values were not associated with ΔMMSE in any subgroup.

**Conclusion:**

Our results suggest a stronger association between NfL levels and cognitive decline in eoFTD than in loFTD, despite generally higher CSF NfL levels in the latter. This may reflect greater neurodegeneration susceptibility or a more direct link with cognitive decline in younger patients. Notably, no associations were found between ΔMMSE and vascular risk factors, microglial activation, or blood‐brain barrier permeability—factors linked to cognitive decline in Alzheimer's disease. These findings highlight potential FTD‐specific mechanisms requiring further investigation to tailor therapeutic strategies

